# Assessment of Olfactory Function in *MAPT*-Associated Neurodegenerative Disease Reveals Odor-Identification Irreproducibility as a Non-Disease-Specific, General Characteristic of Olfactory Dysfunction

**DOI:** 10.1371/journal.pone.0165112

**Published:** 2016-11-17

**Authors:** Katerina Markopoulou, Bruce A. Chase, Piotr Robowski, Audrey Strongosky, Ewa Narożańska, Emilia J. Sitek, Mariusz Berdynski, Maria Barcikowska, Matt C. Baker, Rosa Rademakers, Jarosław Sławek, Christine Klein, Katja Hückelheim, Meike Kasten, Zbigniew K. Wszolek

**Affiliations:** 1 NorthShore University Health System, Evanston, Illinois, United States of America; 2 Department of Biology, University of Nebraska at Omaha, Omaha, Nebraska, United States of America; 3 Department of Neurological and Psychiatric Nursing, Medical University of Gdańsk, Gdańsk, Poland; 4 Department of Neurology, St. Adalbert Hospital, Copernicus PL Sp. z o.o, Gdańsk, Poland; 5 Department of Neuroscience, Mayo Clinic Jacksonville, Jacksonville, Florida, United States of America; 6 Department of Neurodegenerative Disorders, Mossakowski Medical Research Center, Polish Academy of Sciences, Warsaw, Poland; 7 Institute of Neurogenetics, University of Lübeck, Lübeck, Germany; 8 Department of Psychiatry and Psychotherapy, University of Lübeck, Lübeck, Germany; 9 Department of Neurology, Mayo Clinic Jacksonville, Jacksonville, Florida, United States of America; Oslo Universitetssykehus, NORWAY

## Abstract

Olfactory dysfunction is associated with normal aging, multiple neurodegenerative disorders, including Parkinson’s disease, Lewy body disease and Alzheimer’s disease, and other diseases such as diabetes, sleep apnea and the autoimmune disease myasthenia gravis. The wide spectrum of neurodegenerative disorders associated with olfactory dysfunction suggests different, potentially overlapping, underlying pathophysiologies. Studying olfactory dysfunction in presymptomatic carriers of mutations known to cause familial parkinsonism provides unique opportunities to understand the role of genetic factors, delineate the salient characteristics of the onset of olfactory dysfunction, and understand when it starts relative to motor and cognitive symptoms. We evaluated olfactory dysfunction in 28 carriers of two *MAPT* mutations (p.N279K, p.P301L), which cause frontotemporal dementia with parkinsonism, using the University of Pennsylvania Smell Identification Test. Olfactory dysfunction in carriers does not appear to be allele specific, but is strongly age-dependent and precedes symptomatic onset. Severe olfactory dysfunction, however, is not a fully penetrant trait at the time of symptom onset. Principal component analysis revealed that olfactory dysfunction is not odor-class specific, even though individual odor responses cluster kindred members according to genetic and disease status. Strikingly, carriers with incipient olfactory dysfunction show poor inter-test consistency among the sets of odors identified incorrectly in successive replicate tests, even before severe olfactory dysfunction appears. Furthermore, when 78 individuals without neurodegenerative disease and 14 individuals with sporadic Parkinson’s disease were evaluated twice at a one-year interval using the Brief Smell Identification Test, the majority also showed inconsistency in the sets of odors they identified incorrectly, independent of age and cognitive status. While these findings may reflect the limitations of these tests used and the sample sizes, olfactory dysfunction appears to be associated with the inability to identify odors reliably and consistently, not with the loss of an ability to identify specific odors. Irreproducibility in odor identification appears to be a non-disease-specific, general feature of olfactory dysfunction that is accelerated or accentuated in neurodegenerative disease. It may reflect a fundamental organizational principle of the olfactory system, which is more “error-prone” than other sensory systems.

## Introduction

Olfactory dysfunction is seen in normal aging [1] and diverse diseases including diabetes [2] sleep apnea [3], and the autoimmune disease myasthenia gravis [4]. In particular, it is a common non-motor manifestation in neurodegenerative diseases, including Parkinson’s disease, Alzheimer’s disease, spinocerebellar ataxia and corticobasal degeneration, as well as in frontotemporal dementia (FTD), which is characterized by behavioral, language, and cognitive manifestations, and the form of FTD linked to chromosome 17 in which parkinsonism is prominent, FTDP-17 [5–12]. In genetic and sporadic forms of neurodegenerative disease, olfactory dysfunction sometimes precedes motor or cognitive symptoms and so has been discussed as a biomarker that may improve diagnostic accuracy [13–17]. For example, it is present in REM sleep behavior disorder, which often precedes Parkinson’s disease motor manifestations by at least four years [13,18]. A fuller understanding of both its value and limitations as a biomarker for neurodegenerative disease can be obtained by characterizing the features of olfactory dysfunction in presymptomatic and symptomatic carriers of highly penetrant mutations that cause neurodegeneration [16,17]. Even though there are often relatively few individuals who can be evaluated, since disease in mutation carriers is highly penetrant, and issues of genetic variability are considerably less than in the general population, studying olfactory dysfunction in carriers offers the possibility of unique insights into the progression of olfactory dysfunction during the pre-symptomatic and symptomatic phases of neurodegenerative disease. In addition to defining an understanding of the salient characteristics of olfactory dysfunction relevant to its function as a biomarker, such studies also have the potential to offer insights into the process of olfaction itself.

Here, we follow this rationale and assess olfactory dysfunction in carriers of *MAPT* mutations who develop FTDP-17. FTD is phenotypically and genetically heterogeneous, being associated with mutations in *MAPT*, *GRN*, *TARDBP*, *FUS*, *C9ORF72*, *VCP*, and *CHMP2B* [19]. *MAPT* mutations have been identified in multiple FTDP-17 kindreds [19,20]. Most *MAPT* point mutations cluster in the gene region encoding tau’s microtubule-binding domain. Different mutations can affect alternative splicing, tau-isoform ratios, and/or tau-protein levels and are associated with different clinical, molecular and neuropathological outcomes [21–24]. The p.N279K mutation is among those that affect tau pre-mRNA splicing, which are associated predominantly with gray matter loss in the medial temporal lobe, while the p.P301L mutation is among those that affect tau-protein structure, which are associated with gray matter loss in the lateral temporal lobe and relative sparing of the medial temporal lobe [25]. Because the p.N279K and p.P301L *MAPT* mutations have different phenotypic consequences, evaluating whether olfactory dysfunction differs in carriers of these mutations can provide unique insight into whether and how differences in genotype contribute to the severity and the character of olfactory dysfunction associated with neurodegenerative disease.

Therefore, we characterized the features of olfactory dysfunction in carriers having these two *MAPT* mutations. We sought to address whether there is an allele-specific effect on the onset or severity of olfactory dysfunction, whether severe olfactory dysfunction is a fully penetrant trait prior to symptom onset, whether it typically occurs prior to the onset of cognitive and motor symptoms, its age dependence and rate of progression, whether it is reversible, and whether genetic background influences the pattern of odors able to be identified. We then evaluated two possible explanations for olfactory dysfunction–that it is associated with the inability to identify specific odors, or that it is associated with the inability to identify all odors reliably and consistently. For this, we first analyzed data from the *MAPT* kindreds, and then evaluated whether our findings for this specific issue were generalizable by analyzing data from a cohort with sporadic Parkinson’s disease and a cohort of aging subjects without neurodegenerative disease. Insights into these questions have the potential to significantly impact investigations into the mechanisms that underlie olfactory dysfunction as well as normal olfactory processing.

## Materials and Methods

### Ethics Statement

This study was approved by the Mayo Clinic Institutional Review Boards (Clinical and Genetic Studies of Neurodegenerative Syndromes, Dystonia and Restless Leg Syndrome, IRB 1087–98), the Bioethics Committee of the Medical University of Gdańsk (The role of neuropsychological assessment and neuroimaging in the early detection of frontotemporal dementia and parkinsonism linked to chromosome 17 (FTDP-17), Polish Ministry of Science and Higher Education project: IP 2010 037870, approval: 135/2011), and the Ethikkommision der Universität zu Lübeck (EPIPARK, approvals 09–069, 15–082). Study subjects were evaluated following written informed consent.

### Study Design

**Fig 1:**
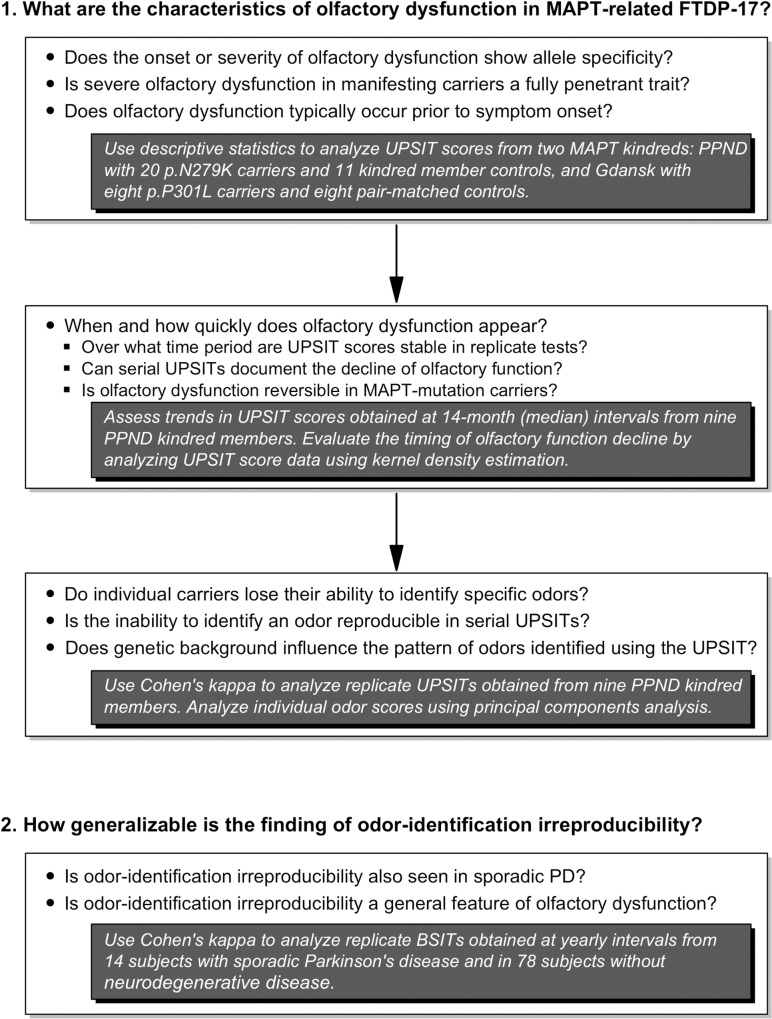
Study questions and analytical approaches.

The questions addressed in this study, and the methods used to investigate them, are diagrammed in the flowchart presented in Fig 1. Our initial focus was to define the characteristics of the onset and progression of olfactory dysfunction in two kindreds with *MAPT*-associated FTDP-17. These were the pallido-ponto-nigral-degeneration kindred (PPND) with the p.N279K *MAPT* mutation, and the Gdańsk kindred with the p.P301L *MAPT* mutation [24,26,27]. The 40-odor forced-choice University of Pennsylvania Smell Identification Test (UPSIT) [28] was used to assess olfactory function, and sex- and age percentile norms based on 1819 men and 2109 women provided with the UPSIT by Sensonics, Inc. were used to assign the percentile associated with a subject’s UPSIT score and to interpret olfactory status as either normosmia, mild microsmia, moderate microsmia, severe microsmia, or anosmia.

Since individuals with these specific *MAPT* mutations are rare, the sample size was limited to the number of kindred members who gave informed consent. In the PPND kindred, we obtained UPSIT data from 20 p.N279K carriers and from 11 mutation-negative kindred members who, since they most closely reflect the genetic background of mutation-positive kindred members, served as controls. In the Gdańsk kindred, we obtained UPSIT data from eight p.P301L carriers (two additional carriers were specifically excluded because of severe cognitive impairment). Eight age- and education-matched subjects were evaluated as controls, as mutation-negative family-member controls were unavailable for testing. An analysis of these data allowed us to address whether the onset or severity of olfactory dysfunction differs between individuals with different mutant *MAPT* alleles, whether severe olfactory dysfunction always occurs, and whether it typically occurs prior to symptom onset.

We then used serial UPSIT administration to determine when and how rapidly olfactory dysfunction occurs. Nine PPND kindred members–eight carriers, one mutation-negative–agreed to participate in this aspect of the study. These nine subjects were assessed two to four times with a median inter-test interval of 14 months (inter-test range: 6 to 34 months). The inter-test interval timing was at least six months to minimize the influence of prior testing. When there was a delay in testing, it was due to the availability of the subject for testing. Replicate UPSIT data are useful for documenting whether replicate UPSITs give stable scores, whether olfactory dysfunction shows any evidence of reversibility, and whether the progression of olfactory dysfunction can be monitored using UPSITs. Analysis of individual UPSIT odors using principal components analysis (PCA) and analysis of the odors identified in replicate UPSITs allowed us to address whether individual carriers lose their ability to identify specific odors, whether the inability to identify an odor in sequential pairs of UPSITs is reproducible, and whether a subset of study subjects exhibit a bias in the pattern of odors they identify using the UPSIT.

Analysis of the results of serial UPSITs in PPND kindred members indicated that the pattern of odors identified in successive UPSITs is often irreproducible (see Results). Therefore, we asked whether odor-identification irreproducibility is also seen in sporadic Parkinson’s disease, and whether it might be a general feature of olfactory dysfunction unrelated to neurodegenerative disease. For this, we retrospectively analyzed differences between annual tests of olfactory function of individuals participating in the EPIPARK study [29]. The EPIPARK study recruited and is following longitudinally 623 members of a representative, population-based cohort from Lübeck, Germany to investigate non-motor symptoms in parkinsonism. In this study, participants were asked to self-report major disease and medical treatments, including medications, socio-economic position, and family history for parkinsonism, and initially underwent transcranial ultrasound of the substantia nigra. Initially and at yearly intervals, the following tests were administered: Mini-Mental State Examination (MMSE) [30,31]; Montreal Cognitive Assessment (MoCA) [32,33]; Parkinson Neuropsychometric Dementia Assessment (PANDA) [34]; Unified Parkinson’s Disease Rating Scale (UPDRS I-IV) [35,36]; major depression questions from the Structured Clinical Interview for DSM IV (SCID) [37]; axis I screening questions from SCID interview; recording of Hoehn and Yahr [38] and Schwab and England activities of daily living scales [39]; Archimedes spiral test and a handwriting sample; and the Brief Smell Identification test (BSIT) [40,41], an abbreviated version of the UPSIT test having a 12-odor subset selected from those in the UPSIT. To address the issue of odor identification irreproducibility, we analyzed BSIT and MoCA data gathered in two successive years from 14 individuals with Parkinson’s disease (an established diagnosis of Parkinson’s disease at each test or at just the second test) and 78 individuals who showed no evidence of neurodegenerative disease. The number of enrolled EPIPARK participants in the Parkinson’s disease and control groups who were twice administered the same version of the BSIT determined by sample sizes of these groups.

In all parts of these investigations, blinding or randomization was impossible given the nature of the study subjects and objectives, however, the clinicians who obtained clinical data and performed olfactory assessment were not involved in data analysis.

### Olfactory testing

The UPSIT was initially administered to 12 p.N279K manifesting carriers (MCs), eight nonmanifesting carriers (NMCs), 11 mutation-negative PPND family member controls, three p.P301L MCs, five p.P301L NMCs, and eight non-Gdańsk-kindred controls. Study subjects provided only general information on their smoking status. Nine PPND individuals–four NMCs, four MC, and one control–underwent additional UPSIT testing at a median inter-test interval of 14 months. Of these, three NMCs did not change disease status in subsequent tests: one had a one duplicate test after nine months; two had three additional tests with inter-test intervals of 14, 15, and 24, or 14, 29, and 10 months. The fourth NMC was symptomatic (i.e., a MC) when a duplicate test was administered 34 months later. Of the four initially tested MCs, three had duplicate tests with inter-test intervals of 6, 9, and 34 months, while one had two additional tests with inter-test intervals of 17 and 28 months. The control individual had a duplicate test after 17 months.

For members of the EPIPARK cohort, we analyzed data from administration of BSITs and MoCAs administered at approximately one-year intervals (13.3 ± 3.4 months). At the second test, the mean age of 78 individuals without evidence of neurodegenerative disease was 67.3 ± 6.5 years and the mean age of 14 individuals with Parkinson’s disease was 70.9 ± 10.4 years. Two of the three available versions of the BSIT were used: 74 subjects, five with Parkinson’s disease, were tested twice using the BSIT; 18 subjects, nine with Parkinson’s disease, were tested twice using version A of the BSIT.

### Statistical analysis

Data were analyzed using non-parametric descriptive statistics, PCA, and kernel density estimation with R [42] or SPSS. The Mann-Whitney U or Kruskal-Wallis tests and the median test were used to evaluate inter-group differences in the distribution and median, respectively, of UPSIT scores. Spearman’s ρ was used to evaluate the strength of the relationship of UPSIT scores to age at test, mutation status, smoking and sex. An alpha-level of *P* < 0.05 was interpreted as significant. Kernel density estimation, using a Gaussian kernel density estimator, was used to estimate the probability density function relating UPSIT scores to age-at-test. PCA was used to evaluate two alternative hypotheses: 1) single or sets of UPSIT odors contributed differentially to UPSIT scores associated with olfactory dysfunction, or 2) odors were equally likely to be misidentified. Cohen’s kappa (κ), an index that measures inter-rater agreement for categorical items and takes into account agreement occurring by chance, was used to assess variability in an individual subject’s olfactory performance on successive UPSITs or BSITs. This measure, which is frequently used to evaluate agreement between different individuals, has also been used to evaluate inter-rater reliability (e.g., [43]) and is useful here since replicate tests of olfactory function made at six month or longer intervals should not be influenced by the memory of prior testing and can therefore be treated as independent assessments. Therefore, κ was used to evaluate how reproducibly a subject identified or misidentified individual odors in successive UPSIT and BSIT assessments.

## Results

### Clinical Information

#### PPND family

**Fig 2:**
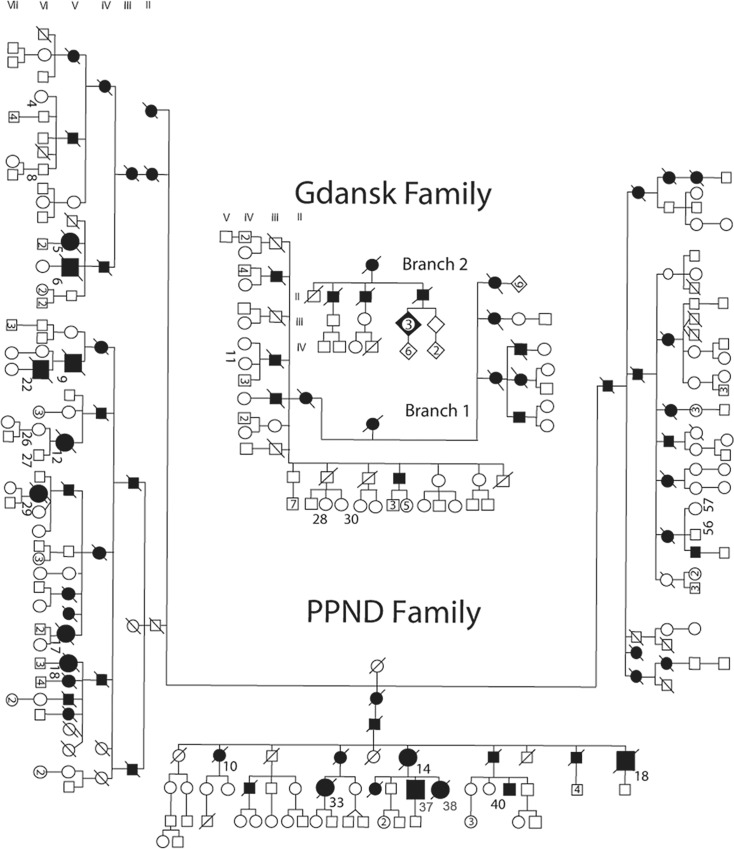
The PPND family and Branches 1 and 2 of the Gdańsk family. Squares, male members; circles, female members; filled symbols; affected mutation carriers; numbers inside symbols, number of siblings; roman numerals, generations; Arabic numerals to the right lower side of a symbol, position in the pedigree, diagonal lines through symbols, deceased. Enlarged symbols identify manifesting carriers in whom UPSITs were administered.

The PPND family is among the largest FTDP-17 kindreds with 57 MCs. Its clinical presentation has been described previously [24–26,44–46], and includes rigidity, bradykinesia, personality changes, dementia, dystonia, gaze palsy and postural instability. The average age of onset in this kindred is 43 years and the average disease duration is eight years. Olfactory function was previously characterized in six affected members [44]. An individual was considered to be an MC, in both the PPND and the Gdańsk kindreds, following the appearance of either a cognitive or motor symptom. Tested MCs are shown as enlarged symbols in the updated pedigree in Fig 2, and clinical and UPSIT data are provided in Table 1. Except for MCs and V-5, the NMC who became symptomatic in the inter-UPSIT interval, tested NMCs and controls are not identified to protect subject confidentiality.

**Table 1:** Clinical information and UPSIT Scores of p.N279K and p.P301L Carriers Identified in Fig 2, and Gdańsk controls.

PedigreeNumber^1^	Sex	Smoker	Status^2^	Age at Onset	Disease Duration at Death	Age at Test	UPSIT Score	UPSIT Norms Percentile^3^	Interpretation^3^
IV-14 (P)	F	Yes	MC	51	6.2	54.3	10	≤5	Anosmia
IV-18 (P)	M	Yes	MC	45	6.2	48.4	11	≤5	Anosmia
V-5 (P)	F	No	NMC	46	2.6	44.8	12	≤5	Anosmia
“ “	“	“	MC	“	“	47.6	11	≤5	Anosmia
V-6 (P)	M	No	MC	41	9.8	42.4	11	≤5	Anosmia
“ “	“	“	MC	“	“	45.2	7	≤5	Anosmia
V-9 (P)	M	Yes	MC	41	9.2	45.6	10	≤5	Anosmia
V-12 (P)	F	No	MC	43	8.0	49.8	11	≤5	Anosmia
V-17 (P)	F	No	MC	39	5.6	41.7	10	≤5	Anosmia
“ “	“	“	MC	“	“	42.4	12	≤5	Anosmia
V-18 (P)	F	No	MC	46	7.1	48.9	10	≤5	Anosmia
V-33 (P)	F	No	MC	48	4.8	51.1	15	≤5	Anosmia
V-37 (P)	M	Yes	MC	47	−	50.8	10	≤5	Anosmia
V-38 (P)	F	Yes	MC	44	3.5	46.6	12	≤5	Anosmia
VI-22 (P)	M	No	MC	38	3.6	39.7	33	12	Mild microsmia
“ “	“	“	MC	38	“	40.2	30	8	Mild microsmia
VI-29 (P)	F	No	MC	39	7.4	41.6	11	≤5	Anosmia
“ “	“	“	MC	39	“	43.0	7	≤5	Anosmia
“ “	“	“	MC	39	"	45.3	8	≤5	Anosmia
III-3 (G)	M	Yes	MC	48	4	52	8	≤5	Anosmia
III-3 (G) control	M	No	Control	−	−	52	36	60	Normosmia
III-4 (G)	M	No	MC	49.5	6.5	56	29	19	Moderate microsmia
III-4 (G) control	M	No	Control	−	−	56	33	27	Mild microsmia
III-5 (G)	M	Yes	MC	46	≤1	46	15	≤5	Anosmia
III-5 (G) control	M	Yes	Control	−	−	47	28	6	Moderate microsmia

^1^P = PPND family, G = Gdańsk family

^2^MC = manifesting carrier, NMC = nonmanifesting carrier, Control = healthy individual pair-matched for sex, age and education

^3^Based on UPSIT age and sex percentile norms provided by Sensonics, Inc.

At the time of their initial UPSIT, the 12 MCs from the PPND kindred (five males, seven females) had a mean age at test of 46.7 ± 4.6 years. Their mean age of onset was 43.5 ± 4.0 years and mean disease duration at test was 3.2 ± 1.4 years. Excluding V-37, a surviving individual, the mean disease duration in MCs was 6.5 ± 2.0 years. At the time of their initial UPSIT, the mean age at test of the eight NMCs and 11 mutation-negative PPND family member controls was 34.2 ± 8.4 and 39.6 ± 14.7 years, respectively.

#### Gdańsk family

The Gdańsk family is the first family from Central-Eastern Europe whose FTDP phenotype associates with the p.P301L mutation [27]. In contrast to previous reports for this mutation, symptomatic individuals from the Gdańsk family show atypical neurological and neuropsychological manifestations, such as unilateral resting tremor and hemi-spatial neglect [47]. Symptoms include behavioral abnormalities and depression. Parkinsonism develops within five years and includes bradykinesia, bradyphrenia, unilateral tremor, postural instability, and falls. With disease progression, epileptic seizures, vertical gaze palsy, and hemispatial neglect are seen. The mean disease duration is ten years.

The Gdańsk family consists of two branches (Fig 2). The individuals tested–five NMCs from Branch I and three male MCs from Branch II–are shown as enlarged symbols. Clinical information on the MCs and their controls are shown in Table 1. Two affected individuals displayed behavioral and cognitive impairment without parkinsonism [27,47].

For MCs, the mean age at test was 51.3 ± 5.0 years, the mean age of onset was 47.8 ± 1.8 years, and the mean disease duration at test was 3.3 ± 4.2 years. The mean age at test of NMCs and the eight pair-matched, mutation-negative individuals was 30.0 ± 2.7 and 37.6 ± 12.1 years, respectively.

### Salient Characteristics of *MAPT*-Related Olfactory Dysfunction

#### Olfactory dysfunction does not appear to be allele-specific

**Fig 3:**
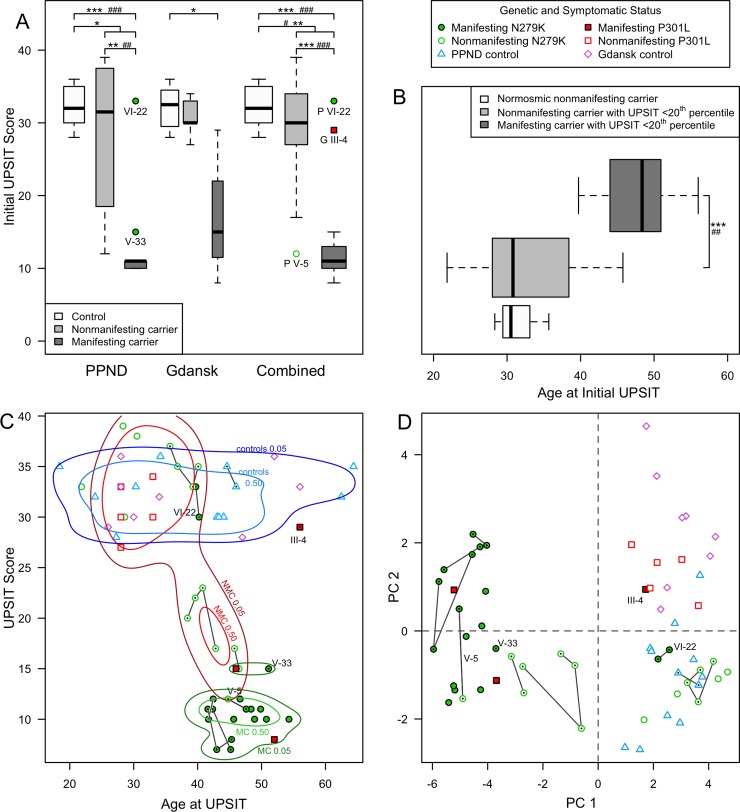
Characterization of olfactory dysfunction in *MAPT* mutation carriers. **(A) UPSIT scores differ between *MAPT* carriers and controls**. The initial UPSIT scores obtained from MCs, NMCs, and familial (for PPND) or pair-matched (for Gdańsk) controls are presented as boxplots showing the medians and interquartile distributions. The boxplot width is proportional to the number of subjects in each group. The main legend shows the symbols that are used throughout this figure to convey genetic and symptomatic status. Here and in panel B, the symbols #, ##, ### and *, **, *** identify differences between groups significant at the *P* < 0.05, 0.01, or 0.001 levels, in either the median (median test) or distribution (Mann-Whitney U or Kruskal-Wallis tests), respectively. Outliers are shown and labeled here and in panels C and D using the pedigree identifiers from Fig 2 and P for PPND or G for Gdańsk. Differences in the distribution or median of UPSIT scores between the controls, NMCs, or MCs of each kindred were not significant, so differences between these groups were also assessed after pooling data from both kindreds. In the combined data, MCs as well as all carriers have different distributions and lower median UPSIT scores than controls, and MCs have a narrower distribution and lower median UPSIT score than either NMCs or controls. **(B) Olfactory dysfunction precedes symptomatic onset in p.N279K and p.P301L carriers.** The initial UPSIT scores of carriers used in part A are replotted as a function of age after grouping carriers by whether their UPSIT score falls below the 20^th^ percentile of UPSIT-score sex and age population norms. In this study, all NMCs above this cutoff were normosmic. Among individuals with UPSIT scores below the 20^th^ percentile, NMCs have a wider age distribution and a younger median age than MCs. **(C) As carriers approach their fourth decade, their UPSIT scores show an accelerated decline. Though UPSIT scores remain relatively stable in replicate tests, individual odors are not identified reproducibly.** UPSIT scores of PPND and Gdańsk kindred members and controls are plotted relative to age at test. The 50^th^ and 95^th^ percentile distribution of kernel density plots for MCs, NMCs, and controls are drawn. These indicate that, except for the outliers identified in Panel A (labeled and marked here with a dot), olfactory function in NMCs, compared to that in controls, declines precipitously near the start of the fourth decade and that MCs have very low (≤15) UPSIT scores early in their fourth decade. Two to four UPSITs were obtained from nine PPND kindred members at a median inter-UPSIT interval of 14 months (inter-test range: 6–34 months; for details, see Methods). Lines connect the data points (marked with a dot) for successive UPSITs in each individual, highlighting that UPSIT scores are relatively stable over the tested intervals. **(D) Principal component analysis using individual odor scores separates study subjects according to affected status and kindred.** The scores of the first and second components from PCA using only individual odor scores, each centered and scaled to have unit variance, are shown in a scatterplot. Outliers and individuals with replicate UPSITs are presented as in panel C. With the exception of two outliers (Gdańsk III-4 and PPND VI-22), PC1 distributes MCs separately from controls, while NMCs are mixed among both controls and MCs. PC2 tends to distribute NMCs and controls, but not MCs, by kindred.

Examination of the distribution of the UPSIT scores, considering only the initial scores of subjects administered multiple UPSITs, from the PPND and Gdańsk families reveals that most MCs have severe olfactory dysfunction, while NMCs have a wide range of UPSIT scores that spans between those of MCs and controls. As shown in Fig 3A, in the PPND family, MCs have a lower median and narrower distribution of UPSIT scores than controls (*P* < 0.001 for both), MCs have a lower median and narrower distribution of UPSIT scores than NMCs (*P* = 0.001 for both), and carriers (MCs and NMCs) show a wider distribution of UPSIT scores than controls (*P* = 0.011). In the Gdańsk family, the distribution of UPSIT scores is wider in MCs than in controls (*P* = 0.024). No significant differences in the median or distribution of UPSIT scores were identified, however, when just MCs, NMCs, or controls from one family were compared to the other. While the small sample size in the Gdańsk kindred may preclude identifying significant differences, these data suggest that olfactory dysfunction is not allele specific.

#### Severe olfactory dysfunction is not a fully penetrant trait

When UPSIT data for MCs, NMCs, and controls from each family are combined, it becomes apparent that even though all MCs have impaired olfactory function, not all have low, or even similar, UPSIT scores. In Fig 3A, the box plots for MCs in the combined data identify two outliers: VI-22 with mild microsmia (UPSIT = 33 at age 39.7, 12^th^ percentile of male norms) and Gdańsk III-4 with moderate microsmia (UPSIT = 29 at age 56, 19^th^ percentile of male norms). Since both of these outliers are non-smokers, and smoking can negatively affect UPSIT scores, one concern is whether these individuals are outliers because they are not smokers while other MCs are smokers. Smoking status seems unlikely to be the only factor, as three relatively young nonsmoker MCs in the PPND family (V-6, V-17, and VI-29, tested at ages 41.6 to 42.4) are anosmic with USPIT scores 10 and 11 (≤ 5^th^ percentile of sex and age norms). Hence, severe olfactory dysfunction is not a fully penetrant trait at the time of symptom onset in MCs with either of these *MAPT* mutations.

#### Olfactory dysfunction typically occurs prior to symptom onset

To evaluate whether olfactory dysfunction typically occurs prior to symptom onset, the ages of MCs and NMCs were compared after grouping them based on whether their (initial) UPSIT score was above or below the 20^th^ percentile of sex and age UPSIT norms. Fig 3B shows the age distribution of these subject groups. The only individuals with UPSIT scores above the 20^th^ percentile were NMCs, who were also normosmic. All MCs had UPSIT scores below the 20^th^ percentile, as did most NMCs. Among individuals with UPSIT scores below the 20^th^ percentile, however, the median age of MCs was higher than that of NMCs (*P* = 0.004), and the distribution of ages of MCs was narrower than that of NMCs (*P* < 0.001). As discussed above, some olfactory function can be preserved in atypical outliers. PPND VI-22 at age 40 had an UPSIT of 30 (8^th^ percentile), while all other p.N279K MCs were over age 41 and anosmic. Gdańsk III-4 at 56 had an UPSIT of 29 (19^th^ percentile). Though older than the other two MCs in the Gdańsk kindred, and having longer disease duration (6.5 versus 0 and 4 years), he had retained greater olfactory function. Nonetheless, substantial olfactory dysfunction occurs in mutation carriers before symptom onset.

#### Olfactory function declines irreversibly and precipitously near the start of the fourth decade

Analyses using initial UPSIT scores showed that they decline strongly with increasing age at test in all carriers and in just PPND carriers (all: Pearson’s *ρ* = −0.723, *P* < 0.01, 2-tailed; PPND: *ρ* = −0.738, *P* < 0.01; Gdańsk: *ρ* = −0.475, *P =* 0.234) but not in any group of controls (all: *ρ* = 0.149, *P* = 0.542; PPND: *ρ* = 0.074, *P* = 0.828; Gdańsk: *ρ* = 0.218, *P* = 0.604). Since smoking is known to influence UPSIT scores, we also assessed the whether UPSIT scores were correlated with smoking in carriers or controls. In contrast to the strong relationship seen between UPSIT scores and age at test in carriers, UPSIT scores showed only negligible or low, but always insignificant correlations with smoking in either carriers (all: *ρ* = −0.161, *P* = 0.414; PPND: *ρ* = −0.323, *P* = 0.165; Gdańsk: *ρ* = −0.063, *P* = 0.881) or in all controls and just PPND controls (all: *ρ* = −0.155, *P* = 0.527; PPND: *ρ* = 0.330, *P* = 0.322). Smoking did a strong and significant negative correlation with UPSIT scores in Gdańsk controls (*ρ* = −0.765, *P* = 0.027), however. We also found that there was no significant relationship between sex and UPSIT in carriers (all: *ρ* = −0.121, *P* = 0.538; PPND: *ρ* = 0.000, *P* = 1.000; Gdańsk: *ρ* = −0.604, *P* = 0.113), or in controls (all: *ρ* = 0.019, *P* = 0.937; PPND: *ρ* = 0.147, *P* = 0.665; Gdańsk: *ρ* = −0.110, *P* = 0.795). These analyses indicate that, while smoking is able to negatively affect UPSIT scores, it does not strongly correlate with the UPSIT scores of the carriers in this study, and so not, by itself, account for the decline of UPSIT scores in carriers. In contrast, increasing age at test strongly and negatively affects UPSIT scores in carriers but not controls.

To better evaluate how the distribution of UPSIT scores changes during aging, non-parametric kernel density estimation, using a Guassian kernel density estimator, was used to estimate the probability density function describing how UPSIT scores change during aging. The 50^th^ and 95^th^ percentile distributions for controls, NMCs, and MCs are drawn in Fig 3C. With the notable exception of the previously discussed outliers, olfactory function in NMCs, which until the late third decade overlaps with that of controls, declines precipitously near the start of the fourth decade so that, at the typical time of symptom onset, MCs have very low (≤15) UPSIT scores.

To evaluate the whether olfactory dysfunction is irreversible, address the stability of UPSIT scores over time, and attempt to follow the timing of the onset and progression of olfactory dysfunction in single individuals, we obtained serial UPSITs from nine PPND kindred members (one gene-negative family member, four MCs, three NMCs and one NMC who manifested symptoms in the interval between two UPSIT administrations) at a median inter-test interval of 14 months. These, together with initial UPSIT scores, are plotted against age at test in Fig 3C by using lines to connect the successive replicate scores obtained from the nine PPND family members. In no case was olfactory dysfunction reversible. Though UPSIT scores in these p.N279K carriers diminish as they approach their fourth decade, the UPSIT scores of individuals with replicate assessments do not show a consistent downward trend over the sampled time intervals. Indeed, the mean of the largest difference between two successive UPSITs in the nine subjects was 3.2 ± 1.5, and UPSIT scores were also stable in two of nine individuals where four UPSITs were obtained over a period of 53 months. The stability of UPSIT scores, even in subjects followed close to the age when carriers as a group show declining UPSIT scores, suggests that it may be challenging to use serial UPSIT testing in a single individual to precisely define the progression of olfactory dysfunction.

#### Olfactory dysfunction affects all odors: odor identification is irreproducible

**Fig 4:**
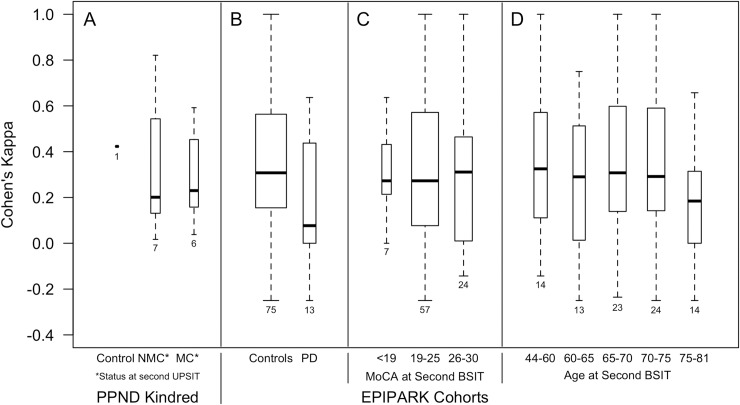
The inability to reliably and consistently identify odors is a general characteristic of olfactory dysfunction. Nine members of the PPND kindred were administered two or more UPSITs at a median inter-test interval of 14 months. 78 EPIPARK subjects without neurodegenerative disease (controls) and 14 EPIPARK subjects with sporadic Parkinson’s disease were administered two BSITs approximately one year apart. Boxplots indicating the median and interquartile distribution of κ, which provides a measure of inter-rater agreement and takes into account agreement occurring by chance, is shown for these groups (A and B). The width of the boxplots is proportional to the number of individuals in each group, noted below each boxplot. The scores of four EPIPARK subjects are not plotted (see text). Study subjects show a range of κ values, but in all groups, the majority of individuals have κ < 0.40, indicating poor to fair agreement. That is, the set of odors that are incorrectly identified on one test shows only poor to fair agreement with the set incorrectly identified on a second test one year later, even in instances when UPSIT or BSIT scores are similar on both tests. In the EPIPARK cohorts, cognitive status, by itself, and age, by itself, do not explain a subject’s inability to reliably and consistently identify specific odors, as when the distribution of κ in all study subjects is grouped by performance on the MoCA (C) or age (D), a range of κ values is seen in all groups.

We also used the replicate UPSIT data in PPND kindred members to address two additional issues: whether individual carriers lose their ability to identify specific odors, and whether their inability to identify an odor is reproducible in successive UPSITs. We used Cohen’s kappa (κ) as a measure of how well an individual subject could identify the same odor in replicate UPSITs. Here, κ is used to provide a measure of intra-rater agreement that takes into account agreement occurring by chance. Values of κ go up to one with values closer to one indicating better agreement. Smaller values are more subject to bias. While specific benchmarks for categorizing values are arbitrary [48–50], a previously described categorization of levels of agreement based on κ values offers a useful perspective [51]: values that are negative or equal to zero indicate poor agreement, 0.01 to 0.20 slight, 0.21 to 0.40 fair, 0.41 to 0.60 moderate, 0.61 to 0.80 substantial, and 0.81 to 1 almost perfect. Fig 4A presents box plots showing the distribution of κ values seen in 14 pairs of successive UPSITs. Moderate or better agreement existed only when UPSIT scores were relatively high or quite low: specifically, in a control and a NMC (UPSIT scores 33–37), and in two MCs (UPSIT scores 7 to 12). The latter two subjects consistently identified only *gingerbread*, however, and only poor or slight agreement existed in all other carriers. In particular, the replicate UPSITs of PPND VI-22, the affected carrier having outlier UPSIT scores of 33 and 30 on successive UPSITs, showed poor agreement (κ = 0.037). We infer that, even though UPSIT scores are relatively stable, the set of odors that are unable to be identified in an individual p.N279K carrier is neither specific nor reproducible over time.

To obtain additional support for the view that odor misidentification in carriers is not specific to one or a set of odors, and that odor identification is irreproducible, we used PCA to evaluate whether there was any bias in the set of odors able to be identified using the UPSIT, and also, whether subsets of study subjects showed a bias in the pattern of odors they identified using the UPSIT. PCA using individual odor scores, each centered and scaled to have unit variance, but not using gender, age, smoking, mutation and disease status, identified distinct patterns in olfactory dysfunction. Although 12 principal components (PCs) are needed to account for 76% of the total variance, PC1 (32.7% of variance) and PC2 (6.3% of variance) can separate study subjects (Fig 3D).

With the exception of the previously discussed outliers, PC1 distributes MCs away from controls, while NMCs are mixed across the range of controls and MCs (Fig 3D). To evaluate whether the separation of carriers with olfactory dysfunction by PC1 is the result of their being unable to reliably identify specific odors in the UPSIT, the PC1 loadings (regression weights) were examined. All are modest (mean 0.152, range 0.047–0.225), and they are distributed uniformly, with half over and half under 0.158, the value expected if each of the 40 different UPSIT odors has an equal contribution. This is the pattern that would be expected if individuals with olfactory dysfunction are unable to reliably identify all, not just a subset of, individual odors. Together with the results of analyzing serial UPSITs using κ, this supports the view that olfactory dysfunction affects all UPSIT-odor classes and its onset is accompanied by increased variability in the set of odors an individual identifies.

#### Kindred-specific odor identification bias

PC2 tends to distribute NMCs and controls, but not MCs, by kindred (Fig 3D). UPSIT odors contributing to PC2 seem related to a subset of nodes within the human odorome network [52]. The odors with appreciable positive loadings [*pizza* (loading = 0.24), *paint thinner* (0.18), *cheddar cheese* (0.17) and *natural gas* (0.16)] seem related to the nodes *odorless-cheese-sour* and *medicine-phenol-harsh*. In the odorome network, these nodes connect to *fruity-sweet-fat-wax*, which connects to *powerful-lilac*. These two nodes seem related to odors with appreciable negative loadings [*grape* (-0.18), *grass* (-0.19), *clove* (-0.20), *licorice* (-0.25), *fruit punch* (-0.29), *lime* (-0.30), *mint* (-0.40), and *lilac* (-0.42)]. That PC2 tends to distribute NMCs and controls by kindred suggests that an additional factor or factors can bias the pattern of odors identified in individuals without severe olfactory dysfunction. The possible nature of such factors, e.g., genetic background, is considered in the Discussion.

### Odor Identification Irreproducibility Is a General Feature of Olfactory Dysfunction

To evaluate whether the inability to identify odors reliably and consistently using a forced-choice test such as the UPSIT is specific to PPND carriers or is also seen in individuals with sporadic Parkinson’s disease, and in control subjects without neurodegenerative disease, we used κ to evaluate agreement in two BSITs administered about one year apart to 14 individuals with sporadic Parkinson’s disease and 78 individuals with a normal neurological clinical presentation. Fig 4B shows the distribution of κ scores for each group. Four individuals (one with Parkinson’s disease) had either all correct or all incorrect odor identifications (BSITs of 12 (n = 2) or zero (n = 2)) in both of their assessments giving κ = −∞ and so these data are not plotted in Fig 4. All other study subjects showed a range of κ values. In the group with Parkinson’s disease as well as in controls, however, the majority of individuals had κ < 0.40, which indicates fair to poor agreement. Thus, the set of odors that were incorrectly identified on one test did not strongly overlap with the set incorrectly identified on a second test one year later, even in instances when BSIT scores were similar on both tests. Neither cognitive status, as evaluated by performance on the MoCA (Fig 4C) nor age (Fig 4D) explain a subject’s inability to reliably and consistently identify specific odors, as a range of κ values are seen in all cognitive-status and age groups evaluated in this study. We conclude from these data that odor-identification irreproducibility in forced-choice tests of olfactory function such as the UPSIT and BSIT is a non-disease specific, general feature of olfactory dysfunction that is accelerated in *MAPT*-related neurodegenerative disease.

## Discussion

To characterize the salient features of olfactory dysfunction associated with *MAPT* mutations that have different clinical, molecular and neuropathological consequences [19–25], we evaluated UPSITs from MCs and NMCs in FTDP-17 kindreds carrying the p.N279K or p.P301L *MAPT* mutations. While we evaluated of a relatively small number of individuals, some trends are evident. Carriers of both alleles show significant, irreversible age-dependent olfactory dysfunction prior to symptom onset, and allele-specific differences in the severity of olfactory dysfunction are not readily apparent. Strikingly, severe olfactory dysfunction (say, UPSIT ≤ 15) is not a fully penetrant phenotype for either allele. However, the appearance of severe olfactory dysfunction in NMCs appears to be associated with subsequent disease onset. Furthermore, evaluation of p.N279K carriers using serial UPSITs did not reveal a gradual decrease in olfactory function, as UPSIT scores were relatively stable. This may mean that olfactory dysfunction, when measured by the UPSIT, occurs in stepwise decrements that are not easily followed. Nonetheless, as a population, *MAPT* carriers typically develop substantial olfactory dysfunction during a relatively short period–one-to-two years–before symptom manifestation.

Olfactory dysfunction affects the ability to identify all odors in the forced-choice UPSIT, and before it becomes severe, UPSIT odors are identified inconsistently. Consequently, no inferences should be made regarding a subject’s long-term ability to identify an individual odor or subset of odors from the administration of a single UPSIT. To our knowledge, this is the first report of individuals at risk for or with dementia and/or Parkinson’s disease to show irreproducible odor identification in a forced-choice smell test. While the analysis of serial UPSITs in the PPND kindred revealed odor-identification irreproducibility in a forced-choice test as a feature of olfactory dysfunction, this feature is a non-disease specific, general characteristic of olfactory dysfunction. Aging subjects with sporadic Parkinson’s disease, as well as subjects with no evidence of neurodegenerative disease frequently are unable to identify the same set of odors in replicate BSITs carried out about a year apart. Neither cognitive status nor age, by themselves, explain a subject’s inability to reliably and consistently identify specific odors.

### Potential study limitations

One important limitation of this study is its sample size, and the unavailability of mutation-negative controls in the Gdańsk family, which necessitated the comparison of p.P301L mutation carriers to pair-matched controls. Also, while the PPND family is large with many affected and at-risk individuals, replicate UPSITs could be obtained only in nine of the 31 carriers and controls. Still, in each of two NMCs, four tests spanning 53 months had only 6.6% average variation and did not reveal a uniform decline in olfactory function (Fig 3C). This suggests that total UPSIT scores from single tests are reliable measures of olfactory function. Nonetheless, increasing the sample size, in particular assessing a greater number of individuals with the p.P301L, or with additional *MAPT* alleles, and longitudinally assessing NMCs and controls over longer time spans would provide more confidence that *MAPT*-related olfactory dysfunction does not demonstrate allele specificity and also provide more insight into whether the UPSIT is effective at documenting the onset of olfactory dysfunction.

A second limitation is that interpretation of UPSIT questions by a subject may be influenced by different cultural norms. However, previous studies demonstrated that UPSIT score differences were independent of ethnicity and education level [53].

A third concern is that cognitive impairment is often present in FTDP-17, just as it is in other FTD syndromes, and smell identification requires not only primary sensory processing, but also retrieving information about the possible smell source from semantic memory [54]. Since abnormal UPSIT scores could reflect overall cognitive impairment, MCs with severe cognitive impairment were excluded. Furthermore, the presence of a true deficit in odor identification is clear from the deficient performance of NMCs lacking cognitive and motor manifestations. It would be useful to assess semantic memory with respect to olfactory dysfunction in these and other kindreds with inherited parkinsonism/dementia using a uniform neuropsychological assessment.

A fourth concern is that the tests selected to assess olfactory function might bias our results. Other approaches to evaluate olfactory function might reveal additional or different attributes of olfactory dysfunction. For example, odor-identification irreproducibility in successive UPSITs and BSITs could be related specifically to the forced-choice nature of these tests, or more generally, to the imperfect reliability of psychophysical tests of olfaction. Some variance among test items is expected, as the test-retest reliability of the UPSIT is around 0.9 (accounting for ~80% of the variability), while that of the BSIT is around 0.75 (accounting for 56% of the variability) [55]. While tests with more items generally are more reliable and sensitive to olfactory deficits, the mechanism by which olfactory information is processed also may contribute to odor-identification irreproducibility, as discussed in the following section. It would therefore be useful to evaluate this finding using other methodologies to better characterize its underlying basis.

### Potential mechanisms underlying olfactory dysfunction in *mapt* kindreds

The etiology of olfactory dysfunction in neurodegenerative disease remains unclear. While environmental factors have been proposed [56], this study supports a demonstrable role for genetic factors, as age-related olfactory dysfunction clearly is associated with *MAPT* mutations. Discerning which aspects of the mechanism of olfactory perception and the processing of olfactory information are affected in mutation carriers remains challenging due to gaps in our understandings of normal peripheral odor coding and the mechanism for processing information from olfactory perception. Previously published studies have evaluated the consistency and characteristics of odor identification and olfactory function in both sporadic and familial forms of Parkinson’s disease and in other neurodegenerative and neurological diseases (see [5,6,57–62] for review, see also [14,16,17,63–85]). Some studies suggest a diagnostic utility for odor-identification testing, sometimes in concert with imaging-based tests, others argue for using odor-identification tests with a limited subset of odors or raise issues related to predictive reliability. Our findings contribute to this discussion. Some of the inconsistency in odor identification that we observe may reflect variability in how odors are initially detected. At the peripheral level, we know that odors are detected using olfactory neurons that express single odorant receptors [86] and that odors are identified using combinatorial coding after neurons with the same odorant receptor transmit odor detection to a specific set of glomeruli in the olfactory bulb [87]. Inconsistent patterns of odor identification may reflect variation in the spectrum, distribution and density of olfactory receptors coupled with variability in the functional activation of single receptors due to the unequal intensities of odorants presented in different tests of olfactory function, including the UPSIT and BSIT. Not only are odorants often composed of multiple chemical compounds, single odorant receptors respond to multiple chemical compounds and multiple odorant receptors are activated by single chemical odorants [52]. Given the “many-to-many” mapping of odorants to receptors, it would be useful to understand whether single-compound odorants have greater consistency or reliability than multiple-compound odorants, assuming that multiple-compound odorants target a broader array of receptor types.

Since odorant receptor neurons are replaced over periods of weeks to months, and newly born neurons must select among temporarily expressed multiple odorant receptor proteins [88], a related explanation for the onset of inconsistency in the identification of individual odors is that it reflects dynamic processes at the peripheral sensory level. For example, there could be changes in the availability of certain types of odorant receptor neurons, or in the strength of their inputs to glomeruli. This could alter the balance of inputs from the set of different olfactory receptors required to correctly identify an individual odor. At different times, the specific nature of the imbalance could vary, so that an odor misidentified at one time point would be different from an odor misidentified at a later time point. Since odor-identification irreproducibility is also seen in individuals without evidence of neurodegenerative disease, it is noteworthy that this hypothesis does not require a neurodegenerative process, only an alteration to the normal dynamics of the population of olfactory receptor neurons and the strengths of their projections. If such a process occurs in *MAPT* carriers, perhaps it is accelerated or accentuated at an earlier age due to the neurodegenerative process, or contributes to already ineffective or inaccurate downstream processing of the signals provided by odor detection.

The identification of an odor also requires cognitive processing that occurs at multiple levels in the brain, with rich cortical involvement as well as the involvement of subcortical structures with reciprocal connections [89,90]. Timing of neuronal activity in the olfactory bulb can also convey odor information [91]. While visual, auditory, and somatosensory systems employ topographic neural maps as a fundamental mechanism to convert stimulus parameters to neuronal spatial relationships, odor information is not organized topographically in the olfactory system. It has been proposed to be dependent on distributive rather than hierarchical afferent connectivity and modulation from multiple neuromodulatory centers for olfactory processing [92], and as a result, is more “error-prone” than other sensory systems. Substantial to severe olfactory dysfunction in *MAPT* carriers could therefore reflect deficits at one or more of these levels and result in an increase in errors in odor identification. Imaging methods such as diffusion tensor imaging and fractional anisotropy, which assess connectivity patterns [93,94], may help assess the neuronal substrates of olfactory dysfunction [95–99] and delineate mutation-specific effects.

The UPSIT and the BSIT, as forced-choice tests, require a subject to select between four single-word descriptions of an odor. Yet perceived odors, even those associated with single odorant molecules, are often described using multiple terms related to contextual memories, reflecting their perception as an “ensemble of components” [52]. They can be associated with specific experiences or objects through retrieving information from semantic memory. Therefore, the onset of inconsistency in a carrier’s identification of individual odors could also reflect a growing deficit in higher-order processing of sensory information, that is, interpreting the set of presumably balanced inputs from a set of olfactory receptors. Since odor-identification irreproducibility is also seen in a cohort of subjects free of neurodegenerative disease, it may also reflect variation in the inherently error-prone perception mechanism.

### Potential mechanisms underlying biased odor-identification

Especially since odor identification is irreproducible, it is intriguing that PC2 was able to use a subset of odors to separate NMCs and controls by family (Fig 3D). That PC2 does not clearly separate MCs by family likely reflects their uniformly poor performance on the UPSIT, but multiple explanations are possible for why NMCs and controls can be separated by family. This could reflect cultural bias, chance effects from the small sample size, environmental differences, or, since we know that there is variation in the set of functional odorant receptors present in the genome, differences in genetic background unrelated to a *MAPT* allele. Some support for the contributions of genetic background comes from seeing that the controls for the PPND kindred, who were mutation-negative family members, cluster with PPND NMCs. Also, the apparent relationship between UPSIT odors having significant contributions to PC2 and nodes in the human odorome [52] supports the hypothesis that this separation may reflect differences in either the subjects’ odorant receptor repertoire and/or differential connectivity patterns among brain regions involved in olfaction. The influence of shared genetic background on phenotypes in kindreds with mutations causing neurodegenerative disease has been highlighted previously. For example, a large family with a *PINK1* mutation shares a pattern of hyperechogenicity in the substantial nigra [100]. Clustering within a family also is entirely consistent with the recent finding that “olfactory fingerprints”––measures developed from matrices of perceived odorant similarity derived from descriptors applied to individual odorants––can reveal meaningful non-olfactory genetic information as they are related to HLA matching [101].

### Perspective for future research

The analysis of rare kindreds with highly penetrant mutations causing neurodegenerative disease is invaluable for characterizing the development and severity of disease manifestations, many of which, like olfactory dysfunction, have been proposed as biomarkers of a prodromal disease state. Furthermore, longitudinal assessment of rare kindreds with monogenic disease forms can help uncover characteristics such as the odor-identification irreproducibility discovered here, which can be difficult to demonstrate in sporadic disease, whose nature is much more heterogeneous. In addition, using such kindreds to investigate olfactory function along with other biomarkers of disease progression, such as functional imaging, should provide a path towards a more comprehensive understanding of olfaction, as well as how olfactory dysfunction occurs neurodegenerative disease.

## Supporting Information

S1 File Data used in this study. All data underlying the findings presented in this manuscript are available as supporting information, with the exception of information that would allow identification of NMCs and control subjects, and individual members of the EPIPARK cohort, which has been withheld to maintain their confidentiality. These confidential data are available by contacting the corresponding author (KM) and will be released to researchers who meet the criteria for access to confidential data subject to approval of the Mayo Clinic Institutional Review Boards, the Bioethics Committee of the Medical University of Gdańsk, and/or the Ethikkommision der Universität zu Lübeck.
(XLSX)
